# CRISPR-Cas in actinomycetes: still a lot to be discovered

**DOI:** 10.1093/femsml/uqaf010

**Published:** 2025-06-12

**Authors:** Lena Mitousis, Ewa Musiol-Kroll, Wolfgang Wohlleben

**Affiliations:** Interfaculty Institute for Microbiology and Infection Medicine Tübingen (IMIT), Microbiology/Biotechnology, University of Tübingen, Auf der Morgenstelle 28, 72076 Tübingen, Germany; Cluster of Excellence, “Controlling Microbes to Fight Infections” (EXC2124), University of Tübingen, 72076 Tübingen, Germany; Interfaculty Institute for Microbiology and Infection Medicine Tübingen (IMIT), Microbiology/Biotechnology, University of Tübingen, Auf der Morgenstelle 28, 72076 Tübingen, Germany; Interfaculty Institute for Microbiology and Infection Medicine Tübingen (IMIT), Microbiology/Biotechnology, University of Tübingen, Auf der Morgenstelle 28, 72076 Tübingen, Germany; Cluster of Excellence, “Controlling Microbes to Fight Infections” (EXC2124), University of Tübingen, 72076 Tübingen, Germany; German Center for Infection Research (DZIF), Partner site Tübingen, 72076 Tübingen, Germany

**Keywords:** CRISPR-Cas, actinomycetes, *Streptomyces*, natural products, antibiotics, beyond-immunity

## Abstract

Actinomycetes are important producers of valuable natural products that are applied in medicine or industry. The enzymes necessary for the synthesis of those compounds are encoded in biosynthetic gene clusters (BGCs) in the genome. However, the discovery of new natural products or the improvement of production levels can be hindered by difficulties in genetic manipulation, since standard methods often do not or not efficiently work in actinomycetes. One possible explanation for this could be the presence of nucleic acid defense systems such as CRISPR-Cas. Even though there is a lot of research published about CRISPR-Cas systems in general, the knowledge about the function of CRISPR-Cas in actinomycetes is very limited. Based on sequence data it is known that CRISPR-Cas systems occur in around half of all sequenced actinobacterial genomes. Moreover, *in silico* analyses of those systems have led to the discovery of new subtypes. The few examples of experimental evidence of CRISPR-Cas activity *in vivo* or *in vitro*, however, point to some special features, regarding crRNA maturation or life-cycle dependent CRISPR-Cas activity. This short review draws attention to this neglected research area and highlights the available data about CRISPR-Cas in actinomycetes.

## Teaser

There is only very limited knowledge about the functions of CRISPR-Cas (immunity and beyond) in the important group of natural product producing actinomycetes

## Introduction

**Figure 1.**
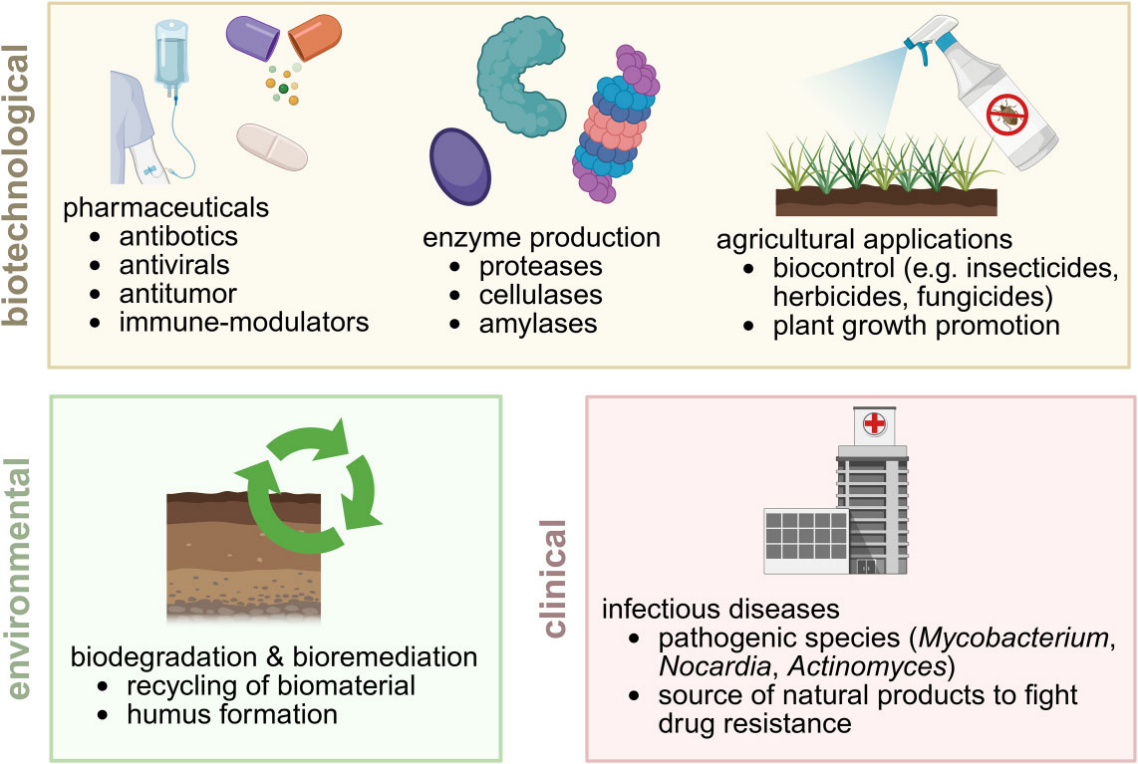
Biotechnological, environmental, and clinical relevance of actinomycetes. Created in BioRender. Musiol-Kroll 2025  https://BioRender.com/0y50xdr.

Bacteria of the class *Actinomycetia* (Salam et al. 2020), also called actinomycetes, belong to one of the most abundant bacterial phyla in nature. They are Gram-positive, GC-rich, and mostly aerobic with a filamentous growth and complex life cycle. Actinomycetes are often found in soil, but they also occur in other ecological niches, such as aquatic habitats. In soil, actinomycetes are involved in the formation of humus and recycling of biomaterials (Fig. 1), as they are able to degrade the remains of plants, animals, and fungi (Saini et al. 2015, Bhatti et al. 2017). Also the characteristic earthy smell of soil and forests caused by the volatile compound geosmin is attributed to actinomycetes, especially the genus of *Streptomyces* (Gerber and Lechevalier 1965). Actinomycetes are also involved in more complex biological interactions, such as the symbiosis between nitrogen-fixing *Frankia* and plant roots (Sellstedt and Richau 2013, Dahal et al. 2017). Associations between antibiotic producing species and insects have also been observed (Kaltenpoth 2009). Most of the actinomycetes are harmless species, but there are also some pathogenic examples (Fig. 1). These include *Corynebacterium diphtheriae* or *Mycobacterium tuberculosis*, which are the cause for the serious diseases diphtheria and tuberculosis, respectively.

CRISPR-Cas systems are known as prokaryotic immunity systems, which sequence-specifically recognize and cleave mobile genetic elements. Additionally, there have been more and more reports about functions beyond immunity, such as involvement in regulation of pathogenicity and virulence (Jones et al. 2012), regulation of cell physiology (Viswanathan et al. 2007), and in DNA-repair (Babu et al. 2011). This has been thoroughly reviewed elsewhere (Faure et al. 2019a, Westra et al. 2014, Devi et al. 2022).

A CRISPR-Cas system consists of the *cas* genes and CRISPR arrays, which contain the repeat sequences with spacers between them. Those spacers are pieces of foreign nucleic acids and are used to specifically target mobile genetic elements. Based on sequence similarities, locus organization and functionality CRISPR-Cas systems are currently divided into 2 classes, 6 types, and 33 subtypes (Makarova et al. 2020). Class 1 is characterized by an effector module consisting of multiple Cas proteins, while class 2 systems have one multisubunit protein as effector. Class 1 includes type I, III, and IV and class 2 type II, V, and VI. The different types are characterized by their specific key enzymes, operon organization, and functionalities. The signature genes for each type are *cas3* for type I, *cas9* for type II, *cas10* for type III, *csf1* for type IV, *cas12* for type V, and *cas13* for type VI. The operon organization of type IV for example is distinct from the others, as it lacks adaptation modules and in some cases also the genes for nucleases. Most CRISPR-Cas types target DNA, but type III can target both RNA and DNA and type VI exclusively targets RNA. The evolutionary classification of CRISPR-Cas systems has been described in detail by Makarova et al. (2011, 2015, 2020) and is regularly updated.

A very important milestone in CRISPR-Cas research was reached, when the Cas9-based genome-editing tool was published (Jinek et al. 2012). It enables precise cutting of target DNA and incorporation of desired modifications in a fast and inexpensive fashion. Since then, a multitude of different versions and types of applications have been derived from it, including systems that have been specifically optimized for the use in actinomycetes. This has already been reviewed in details elsewhere (Alberti and Corre 2019, Tong et al. 2019, Mitousis et al. 2020, Heng et al. 2021, Lee et al. 2024, Wang et al. 2024).

Even though there is a tremendous amount of research about the natural function of CRISPR-Cas systems in bacteria and archaea, CRISPR-Cas of actinomycetes remain unexplored. Because of this, a compact summary of the information on CRISPR-Cas systems in actinomycetes that is available in the literature is provided here in this short review.

### Actinomycetes are an important source for natural products

The main research interest in actinomycetes lies in their potential to produce a diverse variety of secondary metabolites. Those encompass various different chemical structures such as macrolides, tetracyclines, β-lactams, glycopeptides, or aminoglycosides. They cover many valuable modes of actions like antibiotic, fungicide, antiviral, insecticide, antitumor, herbicide, or immune-modulation (Fig. 1). About 60% of the antibiotics used today are originally derived from actinomycetes compounds (Bérdy 2012). The genes for the synthesis of those compounds as well as regulator and resistance genes are often next to each other and coexpressed from biosynthetic gene clusters (BGCs). On average, there are 16 BGCs in one actinomycetes genome but there are also genomes that encode more than 60 BGCs (Seshadri et al. 2022). So-called “silent BGCs,” which are not expressed under standard laboratory conditions, occur frequently. To exploit the available genetic potential (e.g. activation of the “silent BGCs”), strategies for the genetic manipulation of those strains are required. Often the standard molecular genetic methods do not work efficiently in nonmodel actinomycetes strains, which leaves many “silent BGCs” untapped. A potential cause for this genetic inaccessibility could lie in the presence of nucleic acid defence systems, since actinobacterial genomes encodes on average six defense systems (e.g. restriction modification or CRISPR-Cas) (Georjon et al. 2023).

### Sequence data show CRISPR-Cas is prevalent in actinomycetes

Analysis of all sequenced bacterial genomes revealed that CRISPR-Cas systems (=*cas* genes and CRISPR arrays) are present in 42.3% of all bacterial and 85.2% of all archaeal genomes (Makarova et al. 2020). But CRISPR-Cas systems are not distributed equally throughout the different bacterial phyla. There are some phyla in which those defense systems are completely absent. A study from 2016 analysed metagenome data from groundwater and focused on microbes from lineages without isolated or cultivated representatives. It was shown, that in only 10.4% of those “underrepresented” bacterial genomes CRISPR-Cas systems were present (Burstein et al. 2016). The same study also evaluated the frequency of *cas* operons in complete bacterial genome sequences found in the NCBI database. Close to 300 genomes of Actinobacteria were assessed and ~50% contain a CRISPR-Cas system (Burstein et al. 2016). About two-thirds of those are type I systems, followed by type III and II systems. Another study from 2018 focused on CRISPR loci in *Streptomyces* genome sequences (Zhang et al. 2018a). They found at least one CRISPR array in 65.7% (46/70) of the analysed genomes. Just over half of them also contain *cas* genes, so 37.1% (26 of 70) of *Streptomyces* genomes encode one or more CRISPR-Cas systems. Almost all of them are classified as type I-E systems, two as type I-C, and one as I-G (formerly I-U). Analysis of the 2104 spacer sequences revealed no similarity to any phage or plasmid sequence for 99.5%. Only the remaining spacer sequences could be mapped to *Streptomyces* plasmids or genomes. There is no correlation between the genome size and occurrence of multiple CRISPR-Cas systems (Burstein et al. 2016) or the total number of spacers (Pourcel et al. 2020). All those studies showed that CRISPR-Cas systems are present in many of the sequenced actinomycetes genomes with a similar or even slightly higher proportion compared to all sequenced bacterial genomes.

The online CRISPR-Cas database, CRISPRCasdb (Pourcel et al. 2020), provides a good overview of the CRISPR-Cas systems found in whole genome sequences. It shows, that the well-characterized model strain *Streptomyces coelicolor* M145 for example has no CRISPR-Cas system on its chromosome. However, the linear plasmid SCP1, which is found in the natural isolate [*S. coelicolor* A3(2)], has a short CRISPR array with four spacers but no *cas* genes. There are also actinomycetes examples with a particularly large number of CRISPR-Cas systems, such as *Actinoalloteichus hoggarensis*, which has eight different CRISPR-Cas systems, including five type I-E, one I-B, one I-C, and one III-D. However, it must be emphasized that the previous information on CRISPR-Cas in actinomycetes and the following examples (Table 1) are exclusively sequence-based and there are no experimental studies dealing with their biological function. Actinomycetes genomes can also be a source of novel CRISPR-Cas configurations, as shown in studies of genome sequences from multiple *C. diphtheriae* and *Corynebacterium striatum* isolates (Sangal et al. 2013, Ramos et al. 2023). Since they are known pathogens, a lot of sequence data has been sampled from clinical isolates. Most *C. diphtheriae* strains have a type II-C or I-E system, but also three different new versions were found. One is a version of a II-C system, which has no *csn2* and *cas4* genes needed for spacer acquisition. And two are variants of a type I-E system, one has the CRISPR array inserted between *cas3* and *cse1* genes (type I-Ea) and the other has an unusual gene organization within the *cas* operon (I-Eb) (Sangal et al. 2013). In *C. striatum*, a similar version of a type I-E system has been identified (type I-E’), where the *cas*-gene organization is also divergent and a phylogenetically different *cas1* gene is in the cluster (Ramos et al. 2023). New types of CRISPR-Cas systems were also discovered in marine *Salinispora* strains (Wietz et al. 2014). All 75 analysed genomes harbored type I CRISPR-Cas systems, mostly type I-E, I-C, and I-B, but there were also three different I-U systems (I-U_csb3, I-U_csx17, and I-U_Sa). Since these are purely bioinformatic studies, there is no experimental data on the functionality of these novel CRISPR-Cas configurations.

**Table 1.** CRISPR-Cas systems from actinomycetes.

Organism	Description	Relevance	References
**Sequence based: novel/unusual CRISPR-Cas configurations**			
*Corynebacterium diphteriae*	Variant of II-C system without *csn2* and *cas4* for adaptationVariant of I-E system with CRISPR array between *cas3* and *cse1*Variant of I-E system with unusual gene organization	Pathogen	Sangal et al. (2013)
*Corynebacterium striatum*	Variant of I-E system with unusual gene organization and phylogenetically different *cas1*	Human commensal, opportunistic pathogen	Ramos et al. (2023)
*Salinispora*	Variants of type I systems (I-U) with unusual organization and reduced number of genes	Environmental bacteria, producers of natural products	Wietz et al. (2014)
*Streptomycetaceae* spp.	Variant of I-E system without *cas1* and *cas2* for adaptation, instead *tnsB* and *tnsC* from Tn*7* transposase	Producers of natural products	Faure et al. (2019b)
*Streptomyces* spp.	Variant of I-E system without *cas1* and *cas2* for adaptation and *cas3* for interference, neighbouring NTPases and small membrane proteins	Producers of natural products	Shmakov et al. (2018)
**Experimental: features of CRISPR-Cas activity**			
*Streptomyces sp*. HK1 linear plasmid pSHK1	TypeI-E system, proof of Cas protein interactions, no activity following phage infection or plasmid transformation	Producer of natural products	Guo et al. (2011)
*Streptomyces avermitilis*	Type I-E system, active against plasmids and partially against phages, low adaptation, enhanced activity during/after sporulation	Producer of natural products	Qiu et al. (2016)
*Mycobacterium tuberculosis*	Type III-A system, active, targeting ssRNA, untrimmed crRNAs with secondary structure, metal-ion-dependent Cas6	Pathogen	Wei et al. (2019)
*Bifidobacterium breve*	Type I-C system, active against plasmids	Human commensal	Han et al. (2024)
*Saccharopolyspora spinosa*	Type I-B system, active against plasmids	Producer of natural products	Wang et al. (2025)
*Actinomyces naeslundii*	Type II-C system, Cas9 crystal structure	Pathogen	Jinek et al. (2014)

Another analysis from 2019 highlighted the occurrence of unusual type I-E systems in the family of *Streptomycetaceae* (Faure et al. 2019b). Those systems encode a CRISPR array and the genes for the effector-complex (*cas3*–*cas8e*–*cse2*–*cas7*–*cas5*–*cas6*) without any genes responsible for adaptation (*cas1* and *cas2*). The adaptation genes are replaced by the Tn7-like transposase genes *tnsB* and *tnsC*. In the Tn7 transposon, TnsB interacts with TnsA and forms a TnsAB complex, which recognizes the transposable element sequence and introduces double-strand breaks (Peters 2014). TnsC interacts with TnsAB and the target DNA to promote excision and insertion of the transposon. In the CRISPR-Cas context TnsB and TnsC likely act as alternative adaptation module, as the associated arrays contain more variations in spacer length than usual (Faure et al. 2019b). There are no additional Tn7-like genes or *cis*-acting terminal structures, which flank these loci, suggesting that there is no transposon activity.

*Streptomyces* genomes were also found to be a source for minimal type I-E variants, which are putative defective derivatives of subtype I-E CRISPR-Cas systems (Shmakov et al. 2018). Those variants have no adaptation modules as *cas1* and *cas2* are missing and they lack the *cas3* nuclease for interference. Instead, they are neighboring predicted STAND (signal transduction ATPases with numerous domains) superfamily NTPases and small membrane proteins, which are part of programmed cell death regulation in eukaryotes (Leipe et al. 2004). This led to the hypothesis that these variants could be involved in signal transduction associated with programmed cell death or dormancy (Shmakov et al. 2018, Makarova et al. 2020). Experimental evidence supporting this hypothesis has not yet been provided. Analogous minimal variants of type I subtypes were also detected in other non-*Streptomyces*, so that the classification into a separate subtype has been considered (Makarova et al. 2020). For example, they also occur in Tn7-like transposons (Peters et al. 2017). Using the Tn*6677* transposon from *Vibrio cholerae* it was experimentally shown that these minimal variants are involved in crRNA-dependent transposition (Klompe et al. 2019).

These genome sequence-based reports show that the analysis of CRISPR-Cas systems from actinomycetes can reveal new insights into the diversity of CRISPR-Cas locus organization and classification. But the biological function and significance of these variants is still largely unknown, as no experimental studies on the characterization of these variants have been published. The identification of new CRISPR-Cas subtypes and their potential unknown functions can be interesting, especially with regard to the development of novel CRISPR-Cas-based tools.

### CRISPR-Cas systems in actinomycetes have atypical characteristics

The occurrence of CRISPR-Cas systems in actinomycetes is relatively easy to determine using *in silico* methods based on bioinformatic analysis of sequence data. Proof that those systems are active as adaptive immunity system or in functions beyond immunity can only be provided by experimental characterizations *in vivo* or *in vitro*. However, such experimental evidence for the functionality of these systems has only been published for very few cases to date. To the best of our knowledge, there are only the following examples of experimentally characterized CRISPR-Cas systems from actinomycetes (Table 1).

The first example is the characterization of a system found on the linear plasmid pSHK1 from *Streptomyces* sp. HK1 by Guo et al. (2011). It is a typical I-E system with eight *cas* genes and seven CRISPR arrays, two flanking the genes and five distributed on the plasmid. Protein–protein interaction studies detected interactions between Cas5 and Cse1, Cse2, Cas2, and Cas6 (formerly described as Cas1A, Cas2A, Cas2B, and Cas3B). The native system was cloned onto a chromosome-integrating plasmid and introduced into *Streptomyces lividans* by conjugation. Activity of the system was investigated by infection with *Streptomyces* phage ΦC31 or transformation using a plasmid. No prevention of infection or transformation has been observed (Guo et al. 2011). It is possible that for this study some criteria were not taken into account, of which we know today, that they are relevant. For example, the phage infection and plasmid transformation experiments were performed under primed conditions, i.e. the sequence of four spacers in direct succession was added to the plasmid and phage. However, the spacer sequences were inserted directly one after the other without consideration of a possible PAM (protospacer adjacent motif), which is very unusual. So far, no update on this CRISPR-Cas system, that would present additional experiments including the PAM from the pSHK1 plasmid, has been published. So, it remains unclear if the system is involved in the prevention of phage infection.

In 2016, the first report of an active CRISPR-Cas system from an actinomycetes strain was published (Qiu et al. 2016). It is a type I-E system from *Streptomyces avermitilis*, which is the producer strain of the important pesticide avermectin. The CRISPR-Cas system was shown to be active against plasmids and partially against phages (Qiu et al. 2016) and to use 5′-AAN-3′ as effective PAM (Xie et al. 2025). When infected by phages, the formation of plaques was not completely prevented, but the size of the plaques was reduced. Infection with a lower number of phages (∼400 PFU/ml) was completely defended (Qiu et al. 2016). Adaptation occurs rather infrequently in the *S. avermitilis* system. Even under *cas1* and *cas2* overexpression conditions, less than 20% of the screened colonies incorporated a new spacer. The most interesting observation is that CRISPR-Cas activity seems to be enhanced during or after sporulation as incorporation of new spacers and loss of a target plasmid was observed to be increased in clones which did sporulate—in comparison to the vegetative growing transformants (Qiu et al. 2016). One possible explanation could be that the CRISPR-Cas activity is upregulated in germinating spores, since at this stage the cells might be more exposed to their environment. Further research is required to confirm this hypothesis.

The next example is the type III-A CRISPR-Cas system from *M. tuberculosis*. CRISPR-Cas systems in mycobacteria are widespread and not only present in pathogens, but also in free-living organisms (Singh et al. 2021, Brenner and Sreevatsan 2023). A wide variety of different types can be found in mycobacterial genomes, from type I (I-C, I-E, and I-G) to type III (II-A) and there might even be some type IV systems (but they are difficult to predict and need functional verification) (Brenner and Sreevatsan 2023). First it was believed, that CRISPR-Cas type III-A systems are exclusive to *M. tuberculosis* (Singh et al. 2021), but lately they were also identified in other mycobacteria, such as *Mycobacterium canettii, Mycobacterium heckeshornense*, and *Mycobacterium* SM1 (Brenner and Sreevatsan 2023). However, many mycobacteria closely related to strains containing a CRISPR-Cas system do not harbor one at all (Brenner and Sreevatsan 2023). Or there are species, such as *M. canettii* for example, which show differences in the presence/absence or the type of system even within isolates of the same species. This suggests that horizontal gene transfer might be the primary mechanism of CRISPR-Cas acquisition in mycobacteria (Singh et al. 2021). Thorough analyses of the mycobacterial CRISPR arrays showed high sequence variations in different *M. tuberculosis* isolates, suggesting activity of the system, as new spacers are incorporated and existing ones lost. This was already utilized in 1997 to develop a high throughput method for evolutionary and epidemiological classification of *M. tuberculosis* isolates, which is called “spoligotyping” (=spacer oligonucleotide typing) (Kamerbeek et al. 1997). At that time, the CRISPR arrays were referred to as repetitive elements, as CRISPR-Cas systems and their function as adaptive immunity systems were not yet known. Many years later, experimental studies on the CRISPR-Cas system from *M. tuberculosis* followed. In 2018, Zhang et al. (2018b) discovered during RNAseq experiments, that the transcription of the CRISPR-Cas system is controlled by CnpB. CnpB is an Orn-like oligoribonuclease that can hydrolyze cyclic di-AMP and di-GMP but also nanoRNAs (oligonucleotides of 5 or less nucleotides). It was shown that the regulatory effect is independent of cyclic di-AMP or di-GMP and therefore the authors concluded that CRISPR-Cas gene expression is regulated by an Orn-like activity, which is mediated by nanoRNAs. They also revealed that the system is functional in processing crRNAs. Shortly after that, experimental proof followed that the type III-A system is active and targets ssRNA (Wei et al. 2019). The system has novel features compared to other known type III-A systems regarding the crRNA maturation. Normally type III-A crRNAs are preprocessed by Cas6 and then additionally 3′-trimmed by non-Cas nucleases. The final crRNAs have no secondary structure. The crRNAs in *M. tuberculosis* are only preprocessed by Cas6 without further trimming, resulting in crRNAs of uniform length with a secondary structure that rather resembles a type I mature crRNA (Wei et al. 2019). Furthermore, it was shown that Cas6, whose activity is usually metal-ion independent, seems to be dependent on Ca^2+^ and Mn^2+^ ions for activity in *M. tuberculosis* (Wei et al. 2019). Recent structural studies of the Csm-complex from mycobacterial type III-A systems further confirmed the atypical mature crRNAs (Zhang et al. 2024). The structure of the Csm-complex is characterized by a stoichiometry of Csm1_1_2_5_3_6_4_1_5_1_ and is the largest type III-A complex that has been resolved so far (Zhang et al. 2024). Another study revealed that the cyclase domain of Cas10 in *M. tuberculosis* produces cyclic oligoadenylate second messenger molecules, which interact with the Csm-complex, binding to the Csm6 ribonuclease and thereby activating immunity by RNA cleavage (Grüschow et al. 2019). This is common for type III-A systems and underlines the resemblance between type III CRISPR-Cas systems and cyclic oligonucleotide-based antiphage signaling systems in bacteria (Cohen et al. 2019). A more in depth review about the characteristics of the type III-A CRISPR-Cas system in *M. tuberculosis* has been provided by Hamdi et al. (2023).

Furthermore, there are hints that the CRISPR-Cas system in *M. tuberculosis* is involved in functions that go beyond immunity. It was shown that the Cas proteins are secreted and act as virulence factors inducing the host immune response (Jiao et al. 2021). They trigger the induction of T-cell response and macrophage apoptosis and activate the NF-κB signaling pathway. Cas6 seems to play the most important role, since mice infected with a ∆*cas6* strain show a similar reduced pathology as ones infected with a complete CRISPR-Cas deletion strain (Jiao et al. 2021).

This was followed in 2024 by the characterization of the *Bifidobacterium breve* type I-C CRISPR-Cas system. Bifidobacteria are actinomycetes that colonize the human gut as commensals and are believed to have health-promoting effects (Hidalgo-Cantabrana et al. 2017). The type I-C CRISPR-Cas system of *B. breve* was shown to be active in plasmid interference assays utilizing a 5′-TTC-3′ PAM (Han et al. 2024).

The most recent example is the type I-B CRISPR-Cas system from *Saccharopolyspora spinosa*. This rare actinomycetes is used for the industrial production of the macrolide biopesticide spinosyn (Madduri et al. 2001). The type I-B CRISPR-Cas system of *S. spinosa* was shown to be active against plasmids in plasmid challenging assays. Systematic analysis of potential PAM sequences identified 5′-TCG-3′, 5′-CCG-3′, 5′-TCC-3′, 5′-TCT-3′, and 5′-CCT-3′ as functional PAMs with strong interference ability (Wang et al. 2025).

There is another example of an actinomycetes CRISPR-Cas, whose activity has not been studied *in vivo*, but it was used for structural elucidation. During analysis of the *Streptococcus pyogenes* Cas9, the Cas9 from the type II-C system of *Actinomyces naeslundii* was used to help with the determination of the atomic structure by X-ray crystallography (Jinek et al. 2014). The authors wanted to gain information about the general molecular architecture of Cas9 (Jinek et al. 2014). With this analysis, they were able to identify, that all Cas9 enzymes share a conserved structural core, which includes the nuclease domains, but not the PAM recognition loop. The reason why the Cas9 of *A. naelsundii* was chosen as an additional example was not addressed in the publication.

These few experimentally characterized CRISPR-Cas systems from actinomycetes show, that there are some unique features in actinomycetes regarding the crRNA maturation or adaptation. Discovery of those new features can be helpful for the understanding of the general biology of actinomycetes, but it can also help with advancing CRISPR-Cas as a method to expand the genetic toolbox.

### Endogenous CRISPR-Cas systems as genome editing tool in actinomycetes

The application of CRISPR-Cas as a genome editing tool has become indispensable in today’s research. Even though a plethora of optimized tools is available for the use in actinomycetes, there is still room for further development of the method. One of the latest advances is the use of endogenous CRISPR-Cas systems for genome editing. This has the advantage that the necessary genes are already present in the target strain and only a minimal CRISPR with one spacer and optionally a repair template need to be introduced. This avoids toxicity due to the expression of foreign genes and reduces the size of the introduced plasmids. For actinomycetes without an endogenous CRISPR-Cas system, systems from other actinomycetes can be employed. This has the advantage, that the genes originate from a more closely related organism and are therefore often less toxic. Application of endogenous CRISPR-Cas systems for efficient editing of the own genome has been shown for *M. tuberculosis* (Rahman et al. 2022), *B. breve* (Han et al. 2024), and *S. spinosa* (Wang et al. 2025). The system of *S. avermitilis* has additionally also been applied for editing of other streptomycetes (Zhou et al. 2024, Xie et al. 2025).

In *M. tuberculosis* the endogenous type III-A system was used for gene knock-in and knock-out as well as for single- and multiple-gene RNAi (Rahman et al. 2022). Using this RNAi screening, the authors were able to identify genes involved in the regulation of growth. Those can be used as potential targets for the development of novel drugs against *M. tuberculosis*. In *B. breve* the endogenous type I-C CRISPR-Cas system was harnessed to introduce gene deletions, single-base substitutions, gene insertions and successive gene editing as proof of concept (Han et al. 2024). The endogenous type I-B system of *S. spinosa* was used for multiple gene deletions (Wang et al. 2025). To increase the general transformation efficiency of the strain, CRISPR-Cas was used to delete restriction-modification systems, enhancing the plasmid transformation efficiency significantly. With this improved background, the deletion of the 75 kp spinosyn BGC and gene insertion was possible as proof of concept(Wang et al. 2025).

The system of *S. avermitilis* was repurposed for genome editing of other *Streptomyces* strains, leading to the activation of 13 out of 21 silent BGCs (including linaridins, NRPS, and polyketides) from nine phylogenetically distant strains (Zhou et al. 2024). And targeted chromosomal deletions (from 8 bp to 100 kb) or genomic integrations were successfully implemented in nonmodel (Xie et al. 2025).

All those examples demonstrate, that harnessing the endogenous CRISPR-Cas systems of actinomycetes, can lead to powerful genome editing tools—even for actinomycetes, that are challenging to genetically manipulate. Especially the use of type I systems, as opposed to the established type II systems (Cas9 or Cas12), has proven to be very promising in actinomycetes. The fact, that type I systems are the most abundant type in actinomycetes likely contributes to the increased compatibility.

## Conclusion

The number of CRISPR-Cas identified in actinomycetes clearly demonstrates that actinomycetes are a good source of CRISPR-Cas systems, often showing special features. The available sequence-based data reveal, that CRISPR-Cas occurs in around half of actinomycetes genomes and that they can harbor novel variants of CRISPR-Cas subtypes. However, the biological function of these novel variants has not yet been characterized in detail. There are only few example of experimentally characterized CRISPR-Cas systems from actinomycetes. Some of them showed unusual features regarding crRNA maturation or life-cycle dependent CRISPR-Cas activity. In particular, the life-cycle dependent CRISPR-Cas activity in *S. avermitilis* is especially interesting and needs further investigation, as the complex life cycle is a specific feature of many actinomycetes. Nonimmunity functions have so far only been shown for the system of *M. tuberculosis*, where the Cas proteins also act as virulence factors. More research is needed to gain further information on the biological role (including adaptive immunity and functions beyond) of CRISPR-Cas in the important group of actinomycetes. It would be interesting to find out, if CRISPR-Cas systems also have regulatory roles in actinomycetes, potentially interacting with their secondary metabolism or stress responses. This basic research will provide a better understanding of these organisms. The gained knowledge could then be utilized to further develop CRISPR-Cas based genetic manipulation tools. These might improve the difficulties in the genetic accessibility of many actinomycetes, which often stands in the way of natural product research.
